# Development of hand phenotypes and changes in hand pain and problems over time in older people

**DOI:** 10.1097/j.pain.0000000000000402

**Published:** 2015-10-30

**Authors:** Daniel J. Green, Kelvin P. Jordan, Joanne Protheroe, Danielle A. van der Windt

**Affiliations:** Arthritis Research UK Primary Care Centre, Research Institute for Primary Care and Health Sciences, Keele University, Staffordshire, United Kingdom

**Keywords:** Hand conditions, Pain, Function, Epidemiology, Prognosis

## Abstract

Hand disabilities are frequent causes of pain and disability in older people, yet knowledge regarding the characteristics and patterns of hand pain and problems over time is lacking. The main aim of this study was to identify subgroups of older individuals with distinct presentations (phenotypes) of hand pain and function, investigate how these might change over a 6-year period, and explore what characteristics and factors are associated with long-term status. The study population stemmed from the North Staffordshire Osteoarthritis Project, a large, general population–based, prospective, cohort study of adults aged 50 years and older. Information on hand pain and problems was collected using questionnaires at baseline, 3 years, and 6 years. Overall, 5617 participants responded at all time points and were included in the analysis. Five phenotypes were identified using latent transition analysis (“least affected,” “high pain,” “poor gross function,” “high pain and poor gross function,” and “severely affected”) based on 8 hand pain and functional items. The most common transition between phenotypes was from “high pain” at baseline to “least-affected” group. There was a high level of stability in individuals in the “least-affected” or “severely affected” group at baseline. Individuals with widespread body pain, nodes, sleep problems, and pain in both hands at baseline were more likely to be in a severe hand phenotype at 6 years. The results provide clinically relevant information regarding the pattern of hand pain and problems over time and factors that predict transition to more severe hand phenotypes.

## Abstract 2

Supplemental Digital Content is Available in the Text.

Five clinically meaningful phenotypes of hand pain and problems indicate some pain improvement but little functional improvement over a 6-year period.

## 1. Introduction

Musculoskeletal hand problems are common in the general population aged 50 years and older, with an estimated 1-month prevalence of 47% for hand problems and of 31% for hand pain, with a significant impact on everyday life.^9^ Women and the older people appear especially vulnerable to the effect of hand problems on their daily activities and independence.^6,9,11,37^ Hand problems in older people can be due to a range of conditions, with osteoarthritis (OA) being the most frequent cause of pain and disability. In a community-based study of adults aged 50 years and older, approximately 80% of older people with hand pain attending a research clinic had radiographic change (Kellgren and Lawrence grade ≥2) in one hand or more joints.^19^ However, there is little information on the course of hand pain and functional limitations in the community-based and primary care samples of older people.^12,15^

A study of adults consulting for hand and wrist problems in general practice reported that the main factors that influenced a poor outcome were female gender, older age, symptom duration over 3 months, and lower coping strategies.^29^ However, individuals consulting for hand problems may reflect a population with more severe symptoms, and therefore, a study based in the general population would capture a wider range of hand symptoms severities.^24^ A recent report has also highlighted the need for insights into risk factors for the onset of hand problems, specifically hand OA, and for changes in symptoms over time.^7^

Hand pain and problems in older adults represent a heterogeneous group of conditions with a variable presentation and prognosis.^14^ Therefore, a more adaptive technique that identifies different profiles of hand pain and problems and the ability to move between profiles over time is needed. A potential impact of this would be that clinicians have more knowledge to identify the likely course of pain and functional limitations in patients presenting with hand problems and patients at the risk of poorer trajectories. The main objectives of this study were to identify subgroups of older individuals with distinct presentations (phenotypes) of hand pain and function, investigate how these might change over a 6-year period, and explore what factors, in addition to baseline hand phenotype, are associated with long-term status.

## 2. Methods

### 2.1. Study design and population

This study was conducted using data from the North Staffordshire Osteoarthritis Project (NorStOP), a large, population-based, prospective, cohort study described in detail elsewhere.^32^ Briefly, all individuals aged 50 years and older registered with 8 local general practices were recruited through a 2-stage mailing process. Participants were initially mailed a health survey (HS) questionnaire, which contained information on sociodemographics, general health, physical function, and bodily pain. Those who reported any hand problems or pain in their hands in the previous 12 months were then mailed a regional pain survey (RPS) (if permission for further contact was given), which collected further detailed information on the hand. This process was repeated with the same HS and RPS at 3-year and 6-year follow-ups. Participants who responded at all 3 time points (baseline, 3 years, and 6 years) to the HS (and RPS if sent) were included in the analysis. The RPS included detailed hand items regarding pain, function, and limitations, including the AUSCAN (Australian/Canadian Osteoarthritis Index)^1^ and AIMS2 (Arthritis Impact Measurement Scale).^20^

### 2.2. Item selection

The selection of items for developing the model of hand phenotypes was based on the previous literature^1,7,16,18^ and through consultation with 8 patient representatives with hand pain and problems from the Research Users Group (RUG) at the Keele University. All hand-related items from the NorStOP questionnaires (HS and RPS), which included items from the AUSCAN and AIMS2 were considered potentially relevant for inclusion in the development of the model and were presented to the RUG.^1,20^ Members of the RUG, in pairs, were asked to rank the items to indicate which represented their hand condition the most. Items that were ranked in the top half by 2 sets of pairs or more were considered as potential items for model development using latent transition analysis (LTA) (see Statistical Analysis).

Items were dichotomised to ease interpretation. As most of the items were measured on a 5-point scale, these were dichotomised so that 0 (low) represented “none or mild” and 1 (high) represented “moderate, severe, or extreme” pain or limitations in function. The other item, “pain in both hands,” was dichotomised into no hand pain or pain only in one hand vs pain in both hands. At each stage of the analysis, should any participant state in his or her HS that he or she had no hand pain/problems in the previous 12 months and subsequently were not sent the RPS, his or her responses to hand items in the RPS were imputed to be “0” (to represent “none”).

### 2.3. Predictors of long-term hand phenotype membership

Potential baseline predictors of changes in hand phenotype membership at 6 years were selected based on existing evidence regarding their prognostic value in patients with hand problems.^7,18,29^ Demographic or lifestyle factors included age, gender, living status, employment status, and social class (based on current or most recent job). In addition to this, general health factors were included, such as widespread bodily pain (American College of Rheumatology),^35^ depression (based on the Hospital Anxiety and Depression Scale (HADS)),^38^ body mass index (BMI), sleep problems,^13^ self-reported frequency of general practitioner consultations, and self-perceived general health status (item from Short Form 12).^34^ Specific hand factors included previous hand injury, previous hand operation, excessive use of hands in hobbies or occupation, self-reported presence of nodes, pain duration over past 12 months, pain in both hands (if not included in the final list of items for phenotype development), impact of hand problems compared to others of the same age, and self-reported diagnosis of rheumatoid arthritis. Finally, the self-reported presence of any comorbid condition (at least 1 of the following: high blood pressure, diabetes, heart, or chest problems) was also used as a potential baseline predictor of phenotype membership at 6 years.

### 2.4. Statistical analysis

#### 2.4.1. Latent Transition Analysis

Latent transition analysis was used to define distinct population subgroups (called states or phenotypes) based on the items relating to hand problems collected at baseline, 3 years, and 6 years. The technique classifies individuals into 1 and only 1 phenotype at each time point (based on their average posterior probability (APP) of belonging in each phenotype, described later), and it determines the transition probabilities of individuals changing phenotypes between each time points investigated.^4,5^

#### 2.4.2. Model development

The main aim of the first stage of analysis was to develop a model that clustered respondents into an optimum number of phenotypes representing the most important factors of hand pain and function (including stiffness). This was performed using the following steps:

(1) The LTA approach was applied using all the items and the optimal number of phenotypes identified based on the Bayesian Information Criteria (BIC) (where a lower number is optimal),^26,28^ entropy (a measure of distinction and amount of overlap between the phenotypes; range, 0-1, where a higher number is optimal),^4,27^ size of each phenotype (>5% of the respondents should be in each phenotype in at least 1 period),^25,36^ and the clinical relevance and interpretation of each phenotype
(2) For the optimal number of phenotypes, each item was removed in turn (backward stepwise procedure), and the models compared on fit (BIC/entropy) and interpretation, with the least influential item removed
(3) Steps 1 and 2 were repeated until removing further items provided no further improvement to the model.

The modelling process defines latent phenotypes for each of the time points investigated (here 3 time points), and so an assessment was made as to whether the definition for each phenotype was comparable at each time point. This would then indicate that the hand condition of an individual who remains in the same phenotype over time points could be regarded as stable. Individuals should clearly be classified into a phenotype at each time point. This was assessed using APPs.^2^ Posterior probabilities represent the probability of membership for an individual in each potential phenotype at each time point given their item scores. Participants are allocated to the phenotype for which their probability is highest. Average posterior probability for individuals allocated to a phenotype should be greater than 0.7.^2^

The LTA approach is able to include respondents with missing data. However, for this analysis, respondents were removed from the analysis if they had missing values on more than half of the measures at any time point analysed. A sensitivity analysis was performed using baseline and 3-year data only to investigate whether including individuals who were lost to follow-up at 6 years resulted in alternative definitions of the phenotypes at baseline and 3 years.

#### 2.4.3. Phenotype characteristics

Phenotype labels were derived from the item-response probabilities for each phenotype. Item-response probabilities (range, 0-1) reflect how likely participants in each phenotype are to respond “1” (high) for each item. Therefore, a probability of “1.00” for a particular item reflects that the participants in that phenotype responded high for that item. Item-response probabilities close to 0.5 reflect more uncertainty in defining phenotypes because half of the individuals in that phenotype would be expected to respond high for that indicator, whereas the other half would not. Baseline characteristics of each phenotype were compared. The characteristics included demographic information (gender, age, social class, employment, cohabitation status, and marital status). In addition, general health factors were compared, including HADS anxiety and depression scores, BMI, SF-12 general health, and sleep problems.^13^ Each of these characteristics was compared between phenotypes, using a *t*-test for continuous measures and a χ^2^ test for categorical or ordinal measures. Transition probabilities of movement between phenotypes from baseline to 3 years and from 3 to 6 years were determined.

#### 2.4.4. Baseline predictors of 6-year phenotype membership

To explore the baseline predictors of 6-year phenotype membership in individuals most likely to seek health care, participants classified into a phenotype representing no hand problems at baseline were first removed. Factors significantly associated with 6-year phenotype from univariable analyses were taken forward into a multivariable multinomial logistic regression.

#### 2.4.5. Sensitivity analysis

Restricting the phenotype sample size to a minimum of 5% of participants may potentially prevent additional clinically meaningful groups being identified. In light of this, a sensitivity analysis was performed relaxing this criterion and exploring the impact of this on the identification of further hand phenotypes.

Mplus version 7.11 and STATA version 13.1 were used for analysis.^22,31^ A *P* value of <0.05 was considered statistically significant.

## 3. Results

Of the original 26,129 individuals contacted, 18,497 (71%) responded to the baseline health survey (Supplementary figure 1, available online as Supplemental Digital Content at http://links.lww.com/PAIN/A175); those who did not respond tended to be of male gender and younger.^21^ Overall, 5751 (22.0% of those invited to the study) responded at all 3 time points (baseline, 3 years, and 6 years), with 5617 participants (21.5% of those invited) providing sufficient data to be included in the analysis; 3308 (58.9% of responders) reported hand problems at baseline or at least 1 follow-up time point. The participants who did not respond at all time points were more likely to be of female gender (56.5% vs 54.0%) and older (mean age, 67.8 years and SD = 10.6 vs 62.6 years and SD = 8.2).

### 3.1. Model development

From the 40 items (listed in Supplementary figure 2, available online as Supplemental Digital Content at http://links.lww.com/PAIN/A175) included in the questionnaire at each time point, 11 remained following review and ranking by the RUG. The optimum model had 5 phenotypes of hand pain and problems. Removing items that did not improve the model fit or distinction between phenotypes resulted in a model based on 8 items (Table 1). The definition of each phenotype remained stable for each time point (baseline, 3 years, and 6 years), and there was a high probability of individuals being classified in their allocated phenotype (all APPs ≥ 0.85). A sensitivity analysis on just baseline and 3-year data (therefore including those who did not respond at 6 years) provided a similar model to using everyone available at 6 years.

**Table 1:**
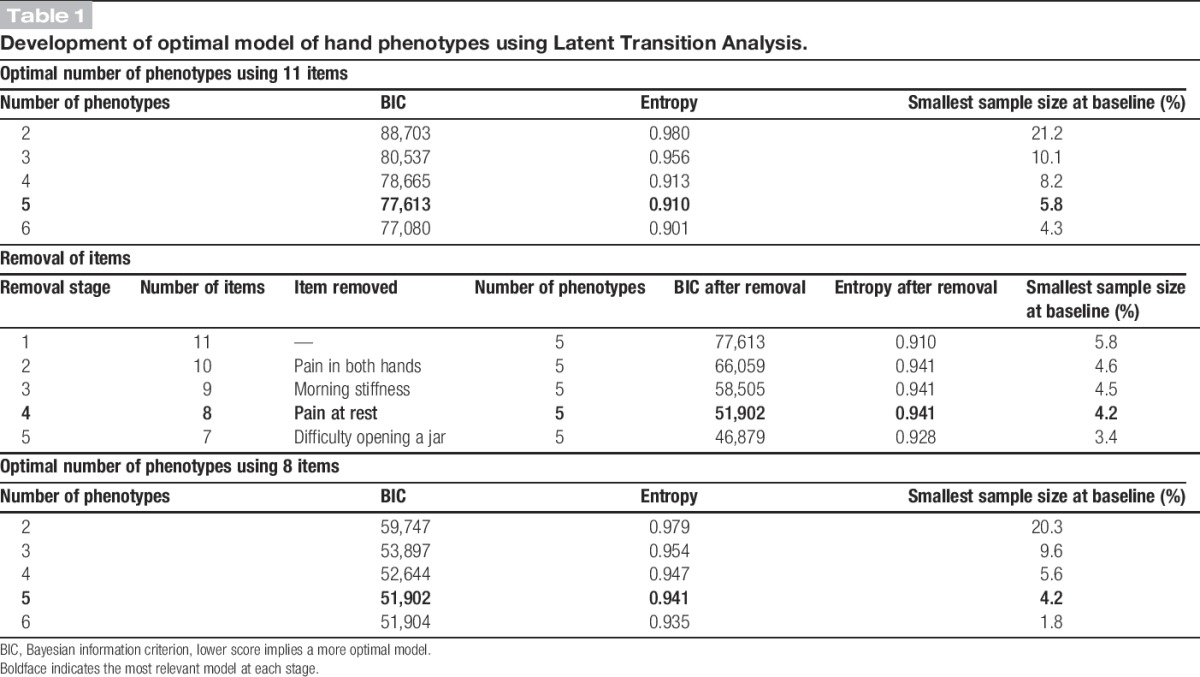
Development of optimal model of hand phenotypes using Latent Transition Analysis.

### 3.2. Phenotype characteristics

The definitions of each of the phenotypes were based on the item-response probabilities displayed in Table 2. The first phenotype (which contained 77% of the population at baseline) was characterised by low probabilities for all of the items, and as such, it was labelled “least affected.” Individuals in the second phenotype (4.3% at baseline) had probability of >0.70 of responding high on the pain items, and they were therefore labelled “high pain. The third phenotype (5.8%) was characterised by high probabilities for 3 functional items (gross functional difficulty) and was labelled “poor gross function.” The fourth group (6.8%) was affected by both pain and problems with gross function, and it was labelled “high pain and poor gross function.” The final group (6.3%) had large probabilities of responding high to all of the items in the model and was therefore labelled “severely affected.”

**Table 2:**
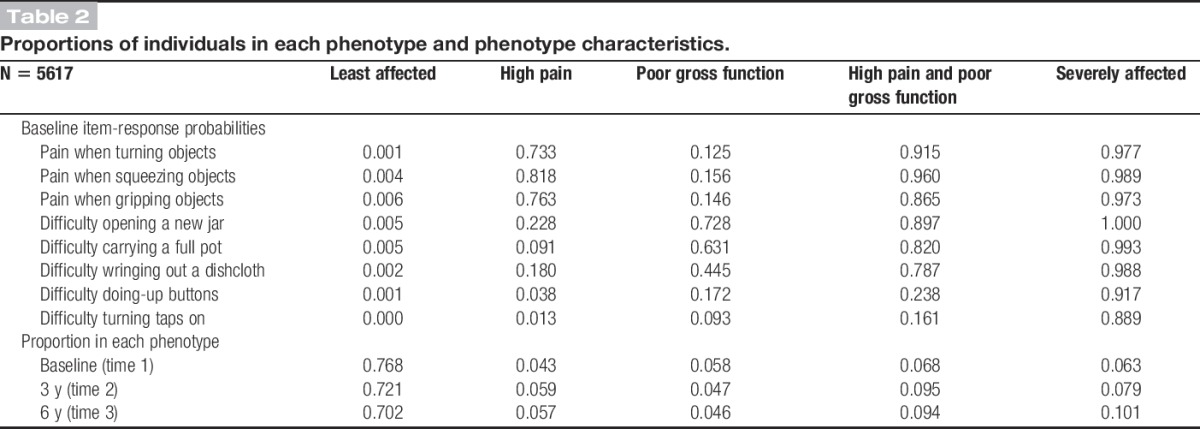
Proportions of individuals in each phenotype and phenotype characteristics.

Participants in the least-affected and high pain phenotypes were more likely to be male, younger, married, have less anxiety and depression, and have (or previously had) a high managerial or professional job compared to the other phenotypes (Table 3); however, those in least-affected phenotype were less likely to have “widespread pain” compared to those in high pain phenotype. Participants in the severely affected phenotype represented a population with more health concerns (higher anxiety, depression, more sleep problems, poorer self-reported general health) along with a larger proportion of female individuals, those who live alone, and older aged compared to the other phenotypes.

**Table 3:**
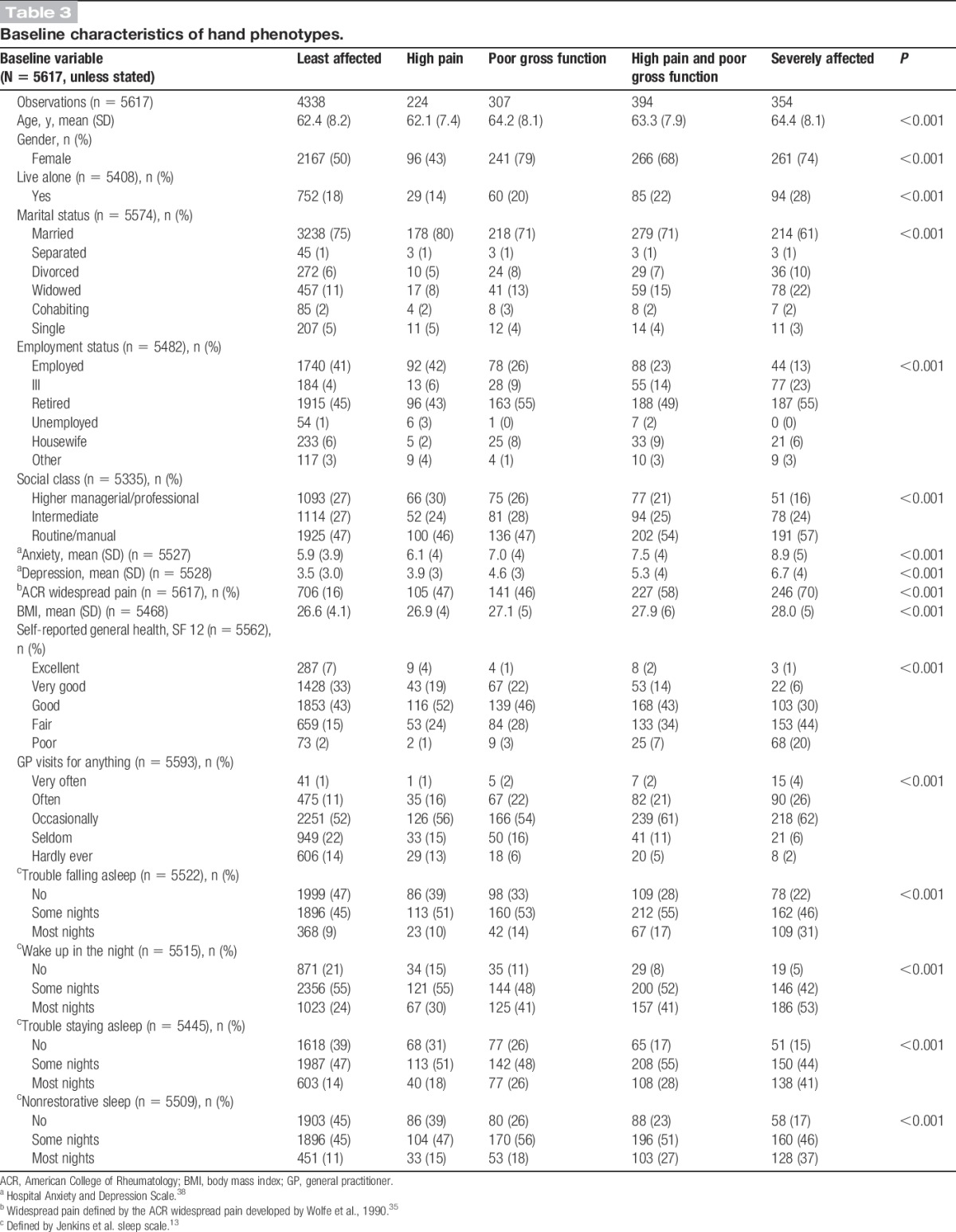
Baseline characteristics of hand phenotypes.

### 3.3. Transitions between time points

There were high levels of stability (remaining in the same phenotype) between baseline and 3 years for individuals in the least affected (87% remained in this phenotype) and severely affected (68%) phenotypes at baseline (Table 4). The largest transitions were seen from individuals moving from high pain phenotype at baseline into the least-affected phenotype at 3 years (42% transitioning). The largest proportion of individuals moving into “severely affected” was from “high pain and poor gross function” (21% transitioning). Overall, 33% of those with poor gross function but not high pain at baseline developed high pain as well at 3 years. Transition probabilities were similar from 3 to 6 years (Table 4).

**Table 4:**
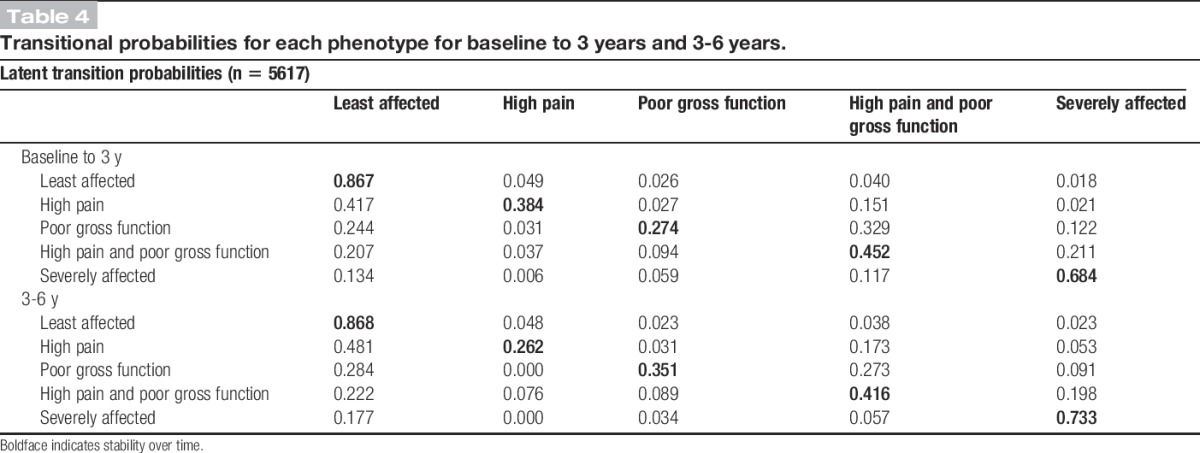
Transitional probabilities for each phenotype for baseline to 3 years and 3-6 years.

### 3.4. Baseline predictors of 6-year phenotype membership

After exclusion of those in the least-affected group at baseline (remaining n = 1025), in the multivariable model (of variables that were significant at the univariable stage), female individuals were significantly more likely to be in the severely affected than in the least-affected phenotype at 6 years (adjusted relative risk ratio [RRR], 1.82; 95% confidence interval, 1.18-2.82), while being male was significantly associated with membership in the high pain state (RRR, 0.54; 95% confidence interval, 0.29-0.97). In addition to this, individuals with sleep problems, presence of nodes, chronic pain duration, pain in both hands, and widespread pain at baseline were more likely to be in more severe hand phenotypes at 6 years (Table 5).

**Table 5:**
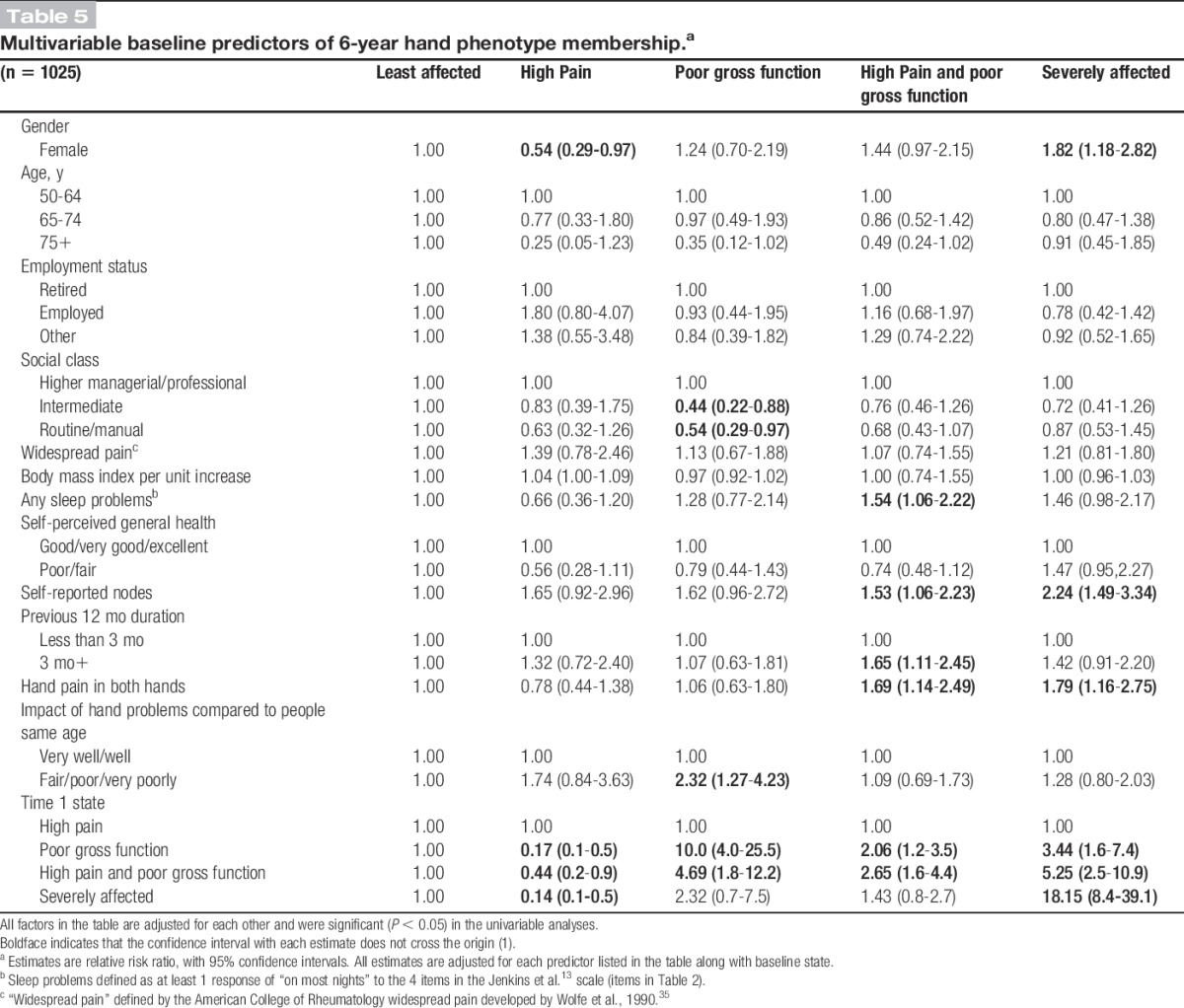
Multivariable baseline predictors of 6-year hand phenotype membership.^a^

### 3.5. Sensitivity analysis

Relaxing the minimum 5% phenotype sample size criterion expanded the LTA model to a 6-phenotype model (Supplementary Table 1, available online as Supplemental Digital Content at http://links.lww.com/PAIN/A175). This additional phenotype (1.8% of the analysis population) had large item-response probabilities for the poor gross function indicators (>0.82) and small for 2 of the 3 pain indicators (<0.20), which reflected a sample of individuals with poor gross function and pain squeezing objects. However, 3 of the 8 indicators had item-response probabilities of around 0.4, which suggested that they did not help to define this phenotype. Therefore, the 5-phenotype LTA model was preferred (Table 2).

## 4. Discussion

This exploratory study has identified 5 phenotypes of hand pain and functional limitations from a population-based sample of older people. Item selection was informed by opinions of older individuals with hand problems. These phenotypes indicate that in general, individuals with functional hand problems are more likely to deteriorate over time, whereas those with hand pain only are more likely to see an improvement in the future. However, once individuals reach the severely affected phenotype (with high probabilities of hand pain and functional limitation), they were less likely to see change over time (stability of >68% at each transition point). An exploratory analysis of predictors of long-term phenotypes suggests that those in the severely affected phenotype at 6 years were more likely to have baseline widespread bodily pain, nodes, and difficulties in sleeping, after adjusting for baseline hand phenotype membership.

### 4.1. Strengths and limitations

The technique of LTA used in this study has some direct benefits for use in musculoskeletal research. The information required for creating the phenotypes was based on a small set of key pain and function items that can be gathered by self-reported questionnaires. In addition to this, the approach of LTA permits individuals to have different profiles of a condition: in this study, levels of pain or types of functional difficulty. Also, it allows individuals to change membership phenotype over time and moves away from presumptions that disease progression advances linearly. There is no universally agreed approach for determining the necessary sample size, but generally, a sample of 200 is needed to perform a reliable basic LTA.^4^ Therefore, this study was of sufficient size to generate reliable results. A limitation of LTA is that there is no gold standard approach to deciding on the number of states. In a sensitivity analysis, we assessed a model with 6 phenotypes and found the additional phenotype to have similarities with another phenotype (high pain and poor gross function) but with some of the items having item-response probabilities around 0.4, suggesting uncertainty in the definition of this new phenotype.

A large proportion of baseline respondents did not respond at all the specified time points, and as such, they were not able to be included in the analysis. It is possible that adults with more severe hand problems or poorer general health were more likely to be lost to follow-up. Although our sensitivity analysis using baseline and 3-year data showed similar phenotype definitions or transitions, this lost to follow-up may have led to an underestimation of the burden and proportion of people with severe hand pain and problem phenotypes in the population. Furthermore, the items analysed in this study were restricted to those collected in the original NorStOP study, and as such, there could be other elements of hand problems that have not been considered, which could alter the profiles of the hand phenotypes, such as Parkinson disease, which was not collected in the NorStOP questionnaires. Because this is a population-based cohort measured at 3-year intervals, it is difficult to be certain what might happen to individuals between the assessment time points and the role any treatment may have had in the course of hand problems.

### 4.2. Relationship with current literature

It is likely that many of the individuals reporting pain and functional difficulty in this study had hand OA. Analysis of a subgroup of participants within NorStOP with additional hand investigations found that of those with hand pain (n = 623), radiographic OA (in 1 or more joints) was present in 78% (n = 485).^17^ That study also showed that other hand conditions were less common (eg, carpal tunnel syndrome, trigger finger, tenosynovitis) and that these were equally distributed across those with and without radiographic change.^17^ As previous research in a primary care–based sample with hand problems has demonstrated that demographic, physical, and psychosocial factors are more strongly associated with hand pain and function outcomes than medical diagnosis,^30^ we assume that the absence of diagnostic information is unlikely to have greatly influenced the resulting functional phenotypes in this study.

It is generally presumed that hand problems in older people are either stable or only progress with more unfavourable outcomes. However, this work has highlighted that although many individuals did remain stable, modest transitions were seen amongst all phenotypes. A large proportion of individuals moved from high pain to least affected (>42%), and even in the more severe phenotype, approximately 30% did transition to other phenotypes. These findings are similar to other trajectory work in other OA locations, such as knee and hip.^3,23,33^ These studies found that groups of individuals did indicate signs of improvement in their OA condition over the study period. One additional benefit of the LTA method used in this study is that it is possible to see in which phenotypes changes are more likely to be expected. Our study found that individuals with functional problems were less likely to improve compared to those with pain only.

There have been limited studies on predictors of the long-term course of hand pain and problems. A previous study in all adults (>18 years) consulting with hand and wrist problems found that factors such as female gender, long symptom duration at presentation, and certain psychosocial factors were predictive of a poorer outcome at 12 months,^29^ similar to the findings in this study. More broadly, a systematic review identified that female gender, age, occupation, pain levels, and personal opinions about hand pain have been shown to be cross-sectionally associated with severity of hand function limitation and hand pain.^24^ These findings are similar to the factors we identified in this study. While previous state membership was, in most cases, the strongest predictor of current state, in addition to the factors listed above, we also found sleep problems, presence of nodes, and bilateral hand pain to be strong predictors of having a more severe hand problem at long-term (6-year) follow-up.

### 4.3. Implications

This exploratory work has defined phenotypes of hand problems, based on self-reported answers to brief pain and functional items. In addition, it provides evidence that there is movement between some phenotypes. Individuals presenting with pain and no functional issues were less likely to get worse over time (and some will improve); however, there was less likelihood of improvement into less severe phenotypes once a member of the “severely affected” phenotype. While this study was exploratory, we have found some evidence that clinicians, particularly those based in primary care, should be aware that those with nodes, sleep problems, and longer duration appear to have an increased risk of worsening hand conditions and may benefit from earlier intervention. In addition, clinicians should be more concerned about older adults consulting for poor hand function because our study has found that they appear to have less chance of recovery and may benefit from self-management approaches, including occupational therapy, joint protection, ergonomic aids, and advice.^8,10^

## 5. Conflict of interest statement

The authors have no conflicts of interest to declare.

The NorStOP study was supported by the Medical Research Council, UK (grant code: G9900220). D.J.G. is funded by a NIHR School for Primary Care Research Doctoral Training Studentship. This report presents independent research commissioned by the NIHR. The views expressed are those of the authors and not necessarily those of the NHS, the NIHR or the Department of Health. DvdW is a member of PROGRESS Medical Research Council Prognosis Research Strategy (PROGRESS) Partnership (G0902393/99,558).

## Supplementary Material

SUPPLEMENTARY MATERIAL

## Appendix A Supplemental Digital Content

Supplemental Digital Content associated with this article can be found online at http://links.lww.com/PAIN/A175.

## References

[1] Bellamy N Campbell J Haraoui B Buchbinder R Hobby K Roth JH MacDermid JC Dimensionality and clinical importance of pain and disability in hand osteoarthritis: Development of the Australian/Canadian (AUSCAN) Osteoarthritis Hand Index. Osteoarthritis Cartilage 2002;10:855–62. 12435330 10.1053/joca.2002.0837

[2] Clark DB Jones BL Wood DS Cornelius JR Substance use disorder trajectory classes: diachronic integration of onset age, severity, and course. Addict Behav 2006;31:995–1009. 16675151 10.1016/j.addbeh.2006.03.016

[3] Collins JE Katz JN Dervan EE Losina E Trajectories and risk profiles of pain in persons with radiographic, symptomatic knee osteoarthritis: data from the osteoarthritis initiative. Osteoarthritis Cartilage 2014;22:622–30. 24662734 10.1016/j.joca.2014.03.009 PMC4028704

[4] Collins LM Lanza ST Latent class and latent transition analysis: with applications in the social, behavioural, and health sciences. New York: John Wiley & Sons, 2010.

[5] Collins LM Wugalter SE Latent class models for stage-sequential dynamic latent variables. Multivariate Behav Res 1992;27:131–57.

[6] Dahaghin S Hand osteoarthritis: epidemiology and clinical consequences [doctoral thesis], The Netherlands: Erasmus University Medical Center, 2005.

[7] Dziedzic K Recent advances in the diagnosis and management of hand osteoarthritis. Int J Clin Rheumatol 2013;8:439–52.

[8] Dziedzic K Nicholls E Hill S Hammond A Handy J Thomas E Hay E Self-management approaches for osteoarthritis in the hand: a 2×2 factorial randomised trial. Ann Rheum Dis 2015;74:108–18. 24107979 10.1136/annrheumdis-2013-203938 PMC4283664

[9] Dziedzic K Thomas E Hill S Wilkie R Peat G Croft PR The impact of musculoskeletal hand problems in older adults: findings from the North Staffordshire Osteoarthritis Project (NorStOP). Rheumatology (Oxford) 2007;46:963–7. 17329350 10.1093/rheumatology/kem005

[10] Hennig T Hæhre L Tryving Hornburg V Mowinckel P Sauar Norli E Kjeken I Effect of home-based hand exercises in women with hand osteoarthritis: a randomised controlled trial. Ann Rheum Dis 2014;0:1–8. 10.1136/annrheumdis-2013-204808 24667900

[11] Hill S Dziedzic K Thomas E Baker SR Croft PR The illness perceptions associated with health and behavioural outcomes in people with musculoskeletal hand problems: findings from the North Staffordshire Osteoarthritis Project (NorStOP). Rheumatology (Oxford) 2007;46:944–51. 17308311 10.1093/rheumatology/kem015

[12] Hill S Dziedzic KS Ong BO Patients' perceptions of the treatment and management of hand osteoarthritis: a focus group enquiry. Disabil Rehabil 2011;33:1866–72. 21859421 10.3109/09638288.2010.550381

[13] Jenkins CD Stanton BA Niemcryk SJ Rose RM A scale for the estimation of sleep problems in clinical research. J Clin Epidemiol 1988;41:313–21. 3351539 10.1016/0895-4356(88)90138-2

[14] Kloppenburg M Kwok WY Hand osteoarthritis—a heterogeneous disorder. Nat Rev Rheumatol 2011;8:22–31. 22105244 10.1038/nrrheum.2011.170

[15] Kloppenburg M Stamm T Watt I Kainberger F Cawston TE Birrell FN Petersson IF Saxne T Kvien TK Slatkowsky-Christensen B Dougados M Gossec L Breedveld FC Smolen JS Research in hand osteoarthritis: time for reappraisal and demand for new strategies. An opinion paper. Ann Rheum Dis 2007;66:1157–61. 17360780 10.1136/ard.2007.070813 PMC1955144

[16] Kwok WY Clinical aspects of hand osteoarthritis: are erosions of importance? [doctoral thesis], The Netherlands: Leiden University, 2013.

[17] Marshall M Patterns of radiographic hand osteoarthritis and associations with pain and function: a prospective cohort study [doctoral thesis], UK: Keele University, 2010.

[18] Marshall M Peat G Nicholls E van der Windt D Myers H Dziedzic K Subsets of symptomatic hand osteoarthritis in community-dwelling older adults in the United Kingdom: prevalence, inter-relationships, risk factor profiles and clinical characteristics at baseline and 3-years. Osteoarthritis Cartilage 2013;21:1674–84. 23954700 10.1016/j.joca.2013.08.004 PMC3819994

[19] Marshall M van der Windt DA Nicholls E Myers H Hay E Dziedzic K Radiographic hand osteoarthritis: patterns and associations with hand pain and function in a community-dwelling sample. Osteoarthritis Cartilage 2009;17:1440–7. 19500560 10.1016/j.joca.2009.05.009

[20] Meenan RF Mason JH Anderson JJ Guccione AA Kazis LE AIMS2: the content and properties of a revised and expanded Arthritis Impact Measurement Scales Health Status Questionnaire. Arthritis Rheum 1992;35:1–10. 1731806 10.1002/art.1780350102

[21] Muller S Thomas E Peat G The effect of changes in lower limb pain on the rate of progression of locomotor disability in middle and old age: evidence from the NorStOP cohort with 6-year follow-up. PAIN 2012;153:952–9. 22386475 10.1016/j.pain.2011.12.006 PMC3355303

[22] Muthén LK Muthén BO Mplus User's Guide. 7th ed Los Angeles: Muthén & Muthén, 1998–2015.

[23] Nicholls E Thomas E van der Windt DA Croft PR Peat G Pain trajectory groups in persons with, or at high risk of, knee osteoarthritis: findings from the Knee Clinical Assessment Study and the Osteoarthritis Initiative. Osteoarthritis Cartilage 2014;22:2041–50. 25305072 10.1016/j.joca.2014.09.026 PMC4256061

[24] Nicholls E van der Windt DA Jordan JL Dziedzic KS Thomas E Factors associated with severity and progression of self-reported hand pain and functional difficulty in community-dwelling older adults: a systematic review. Musculoskeletal Care 2012;10:51–62. 22290761 10.1002/msc.1007

[25] Nylund KL Latent transition analysis: modeling extensions and an application to peer victimization [doctoral thesis], University of California, 2007.

[26] Nylund KL Asparouhov T Muthen BO Deciding on the number of classes in latent class analysis and growth mixture modelling: a Monte Carlo simulation study. Struct Equ Modeling 2007;14:535–69.

[27] Ramaswamy V Desarbo WS Reibstein DJ Robinson WT An empirical pooling approach for estimating marketing mix elasticities with PIMS data. Market Sci 1993;12:103–24.

[28] Sclove SL Application of model-selection criteria to some problems in multivariate analysis. Psychometrika 1987;52:333–43.

[29] Spies-Dorgelo MN van der Windt DA Prins PA Dziedzic KS van der Horst HE Clinical course and prognosis of hand and wrist problems in primary care. Arthritis Rheum 2008;59:1349–57. 18759252 10.1002/art.24019

[30] Spies-Dorgelo MN van der Windt DAWM van der Horst HE Prins AP Stalman WA Hand and wrist problems in general practice- patient characteristics and factors related to symptom severity. Rheumatology (Oxford) 2007;46:1723–8. 17938132 10.1093/rheumatology/kem253

[31] StataCorp. Stata statistical software: release 13. College Station: StataCorp LP, 2013.

[32] Thomas E Wilkie R Peat G Hill S Dziedzic K The North Staffordshire Osteoarthritis Project—NorStOP: prospective, 3-year study of the epidemiology and management of clinical osteoarthritis in a general population of older adults. BMC Musculoskelet Disord 2004;5:2. 14718062 10.1186/1471-2474-5-2 PMC324560

[33] Verkleij SP Hoekstra T Rozendaal RM Waarsing JH Koes BW Luijsterburg PA Bierma-Zeinstra SM Defining discriminative pain trajectories in hip osteoarthritis over a 2-year time period. Ann Rheum Dis 2012;71:1517–23. 22492782 10.1136/annrheumdis-2011-200687

[34] Ware J Jr Kosinski M Keller SD A 12-Item short-form health survey: construction of scales and preliminary tests of reliability and validity. Med Care 1996;34:220–33. 8628042 10.1097/00005650-199603000-00003

[35] Wolfe F Smythe HA Yunus MB Bennett RM Bombardier C Goldenberg DL Tugwell P Campbell SM Abeles M Clark P Fam AG Farber SJ Fiechtner JJ Franklin CM Gatter RA Hamaty D Lessard J Lichtbroun AS Masi AT McCain GA Reynolds WJ Romano TJ Russell IJ Sheon RP The American College of Rheumatology 1990 criteria for the classification of fibromyalgia. Report of the multicentre criteria committee. Arthritis Rheum 1990;33:160–72. 2306288 10.1002/art.1780330203

[36] Yang CC Evaluating latent class analysis models in qualitative phenotype identification. Comput Stat Data Anal 2006;50:1090–104.

[37] Zhang Y Niu J Kelly-Hayes M Chaisson CE Aliabadi P Felson DT Prevalence of symptomatic hand osteoarthritis and its impact on functional status among the elderly: the Framingham Study. Am J Epidemiol 2002;156:1021–7. 12446258 10.1093/aje/kwf141

[38] Zigmond AS Snaith RP The hospital anxiety and depression scale. Acta Psychiatr Scand 1983;67:361–70. 6880820 10.1111/j.1600-0447.1983.tb09716.x

