# Supplementary material for: Development of hand phenotypes and changes in hand pain and problems over time in older people
Source: Pain. 2015 Oct 30;157(3):569–76. doi: 10.1097/j.pain.0000000000000402 (PMC4751742; doi:10.1097/j.pain.0000000000000402)

Supplementary Figure 2

**Initial 40 items considered for analysis and discussed with Research User Group**

AUSCAN (15 items):

- How much pain do you have:
  - At rest?
- How much pain do you have:
  - Gripping objects?
- How much pain do you have:
  - When lifting objects?
- How much pain do you have:
  - When turning objects with your hands?
- How much pain do you have:
  - When squeezing objects with hands?
- How severe is stiffness in hands after first waking in the morning?
- How much difficulty do you have:
  - Turning taps on?
- How much difficulty do you have:
  - Turning a door-knob or handle?
- How much difficulty do you have:
  - Doing up buttons?
- How much difficulty do you have:
  - Fastening jewellery?
- How much difficulty do you have:
  - Opening a new jar?
- How much difficulty do you have:
  - Carrying a full pot with one hand (i.e. a saucepan)
- How much difficulty do you have:
  - Peeling vegetables/fruit?
- How much difficulty do you have:
  - Picking up large heavy objects?
- How much difficulty do you have:
  - Wringing out washcloths (i.e. a flannel)?

AIMS2 (Hand and Finger Function) (3 items):

- Could you easily write with a pen or pencil?
- Could you easily turn a key in a lock?
- Could you easily tie a knot/bow?

AIMS2 (Arthritis Pain) (3 items)

- How often did you have pain in two or more hand joints?
- How often did morning stiffness in your hands last more than one hour from waking up?
- How did your hand problems make it difficult for you to sleep?

AIMS2 (Overall Arthritis Impact) (1 item)

- How often have you taken medication for hand symptoms?

Stand-alone questions (12 items):

- Hand problems in both hands (or one hand only)
- How much hand stiffness do you usually have?
- How much hand aching do you usually have?
- How much hand tenderness do you usually have?
- How much hand weakness do you usually have?
- How much hand clumsiness do you usually have?
- How much burning sensation do you usually have in your hand?
- How much hand tingling do you usually have?
- How much hand numbness do you usually have?
- How often did your hands feel hot or warm?
- How often did your hand problems make you feel frustrated?
- How often did hand problems cause you to drop objects?

Previous hand experiences (5 items):

- Duration of hand pain
- Previous injury to hand
- Operation on hand
- Job that has involved excessive use of hands
- Hobby/pastime that has involved excessive use of hands

Presence of nodes (1 item):

- Finger(s) with nodes


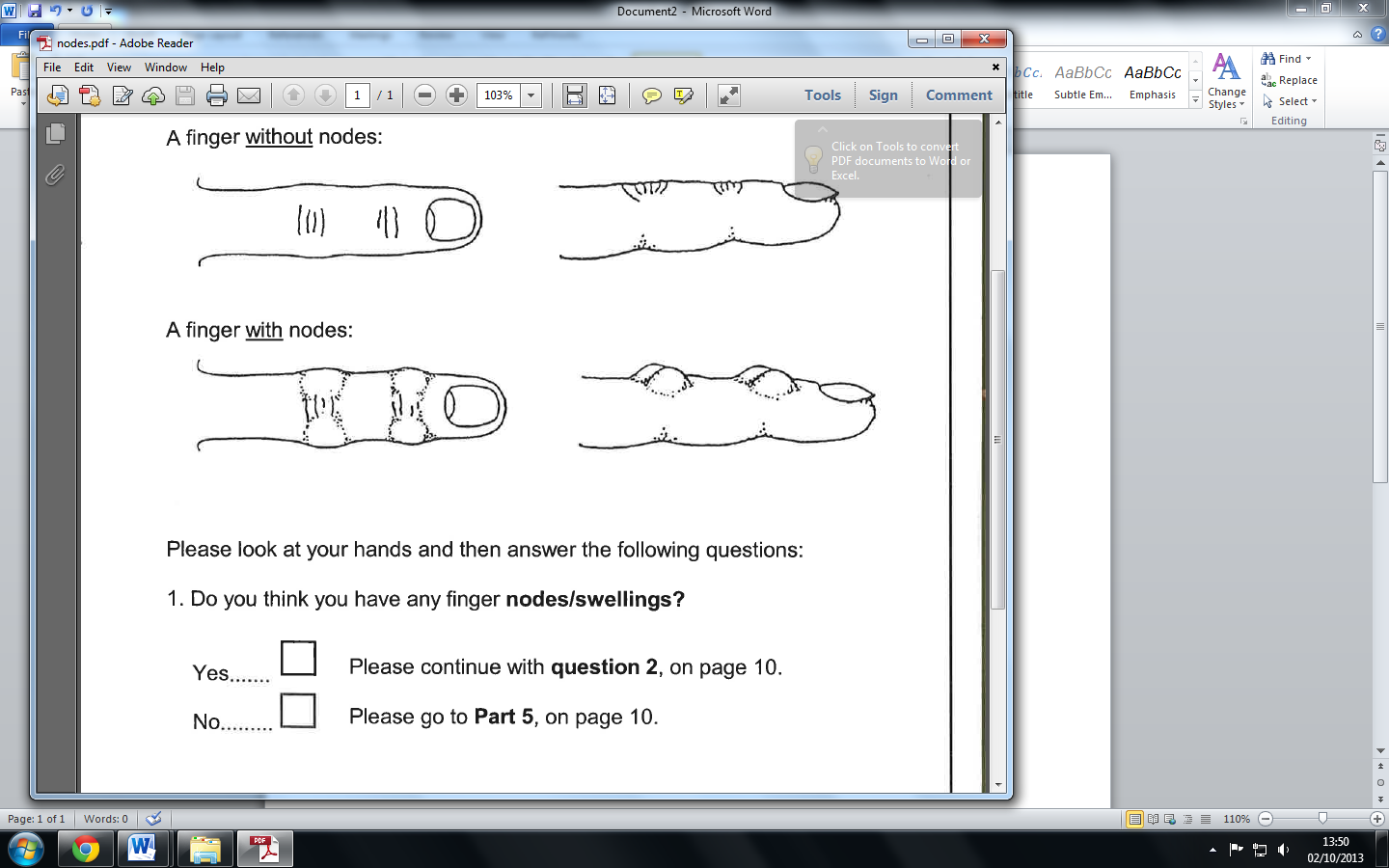

Supplement: SUPPLEMENTARY MATERIAL [file jop-157-569-s001.docx]
